# Coactivation of CB1 and GPR55 promotes GABA release and motor behavior at striatonigral terminals through increased dimerization induced by CB1 activation

**DOI:** 10.3389/fnmol.2026.1717829

**Published:** 2026-02-26

**Authors:** José Arturo Avalos-Fuentes, Rodolfo Sánchez-Zavaleta, Ihosvany Rodríguez Pérez, Rafael Jijón-Lorenzo, Refugio Cruz-Trujillo, María Fernanda González de la Torre, Martha Abigail Villareal Zuñiga, Benjamín Florán

**Affiliations:** 1 Departamento de Fisiología, Biofísica y Neurociencias, Centro de Investigación y de Estudios Avanzados del Instituto Politécnico Nacional, Mexico City, Mexico; 2 Division of Research and Translational Education, Centros de Integración Juvenil, Mexico City, Mexico; 3 Escuela de Ciencias Químicas, Benemérita Universidad Autónoma de Chiapas (UNACH), Ocozocoautla de Espinoza, Mexico

**Keywords:** CB1 receptor, GPR55, heteromers, motor behavior, substantia nigra

## Abstract

CB1 and GPR55 receptors form heteromers in striatal neurons; however, the effects of these heteromers on GABA release at their terminals and their impact on motor behavior remain unknown. In this study, we investigate the presence of CB1-GPR55 heteromers on striatonigral neurons and their axon terminals, and also assess their impact on cAMP accumulation, GABA release, and motor behavior. Furthermore, we explore the effects of sequential receptor activation to examine the phenomenon of increased dimerization induced by receptor activation. A PLA assay combined with Substance P immunofluorescence demonstrated the presence of CB1-GPR55 heteromers in the dorsal striatum and substantia nigra of rats. The kainic acid lesion in the striatum leads to a decrease in PLA dots in both regions. Sequential activation of CB1R, followed by GPR55 activation (CB1→GPR55), increased cAMP accumulation and GABA release at the nigral terminals more compared to GPR55 alone activation. In contrast, simultaneous activation (CB1 + GPR55) or the reverse (GPR55→CB1) did not affect the stimulation effects of GPR55 on cAMP accumulation or GABA release. Additionally, CB1/GPR55 immunoprecipitation in synaptosomes revealed an increase during the sequential activation of CB1→GPR55. Treatments with PTx or ChTx did not alter the effects of CB1→GPR55 sequential activation on GABA release. Finally, intranigral injections of a CB1→GPR55 agonist induced more contralateral turns than GPR55 activation alone. These findings indicate that the sequential activation of CB1→GPR55 within CB1/GPR55 heteromers in striatonigral neurons enhances cAMP accumulation, GABA release, and motor behavior by increasing heteromerization via CB1 activation.

## Introduction

The cannabinoid receptor 1 (CB1) is one of the most abundant G protein-coupled receptors (GPCR) in the brain (Herkenham et al., 1990). Numerous studies indicate their ability to form both homomers and heteromers (Franco et al., 2007), including with orexin receptors (Berrendero et al., 2018; Jäntti et al., 2014; Kim et al., 2021; Ward et al., 2011), CB2 receptor (Callén et al., 2012), adenosine A2A receptor (Carriba et al., 2007; Moreno et al., 2018; Tebano et al., 2012), dopamine D2 receptor (Navarro et al., 2008; Przybyla and Watts, 2010), 5-HT2A receptor (Przybyla and Watts, 2010), μ-opioid receptor (Pickel et al., 2004) angiotensin AT1R receptor (Rozenfeld et al., 2011), and GPR55 (Martínez-Pinilla et al., 2014, 2020a). Several of these heteromers are expressed in the CNS (Callén et al., 2012; Jäntti et al., 2014; Moreno et al., 2018). Significantly, CB1 receptor activation facilitates dimerization with orexin, angiotensin, and D2 receptors (Berrendero et al., 2018; Imperatore et al., 2016; Kim et al., 2021; Przybyla and Watts, 2010; Rozenfeld et al., 2011), indicating that CB1 receptors can promote dimerization and stabilization of heteromers during activation.

Presynaptic CB1 receptors are densely expressed in the substantia nigra (Herkenham et al., 1991; Mátyás et al., 2006), where they inhibit glutamate (Szabo et al., 2000) and GABA release (Chan et al., 1998; Yanovsky et al., 2003), modulating motor behavior (San et al., 1996). In contrast, the orphan GPR55, considered by “cannabinoid receptor type 3” is expressed in striatal medium spiny neurons (Marichal-Cancino et al., 2017; Celorrio et al., 2017; Martínez-Pinilla et al., 2014, 2020a; Ryberg et al., 2007) and in striatonigral terminals of substance P-positive neurons, where it increases GABA release via cAMP/PKA signaling (Sánchez-Zavaleta et al., 2022). This increase in GABA release promotes motor activity following intranigral administration of a GPR55 agonist and occurs independently of dopamine or D1 receptor signaling (Sánchez-Zavaleta et al., 2022).

CB1 and GPR55 receptors are co-expressed in striatal medium-sized spiny neurons (Martínez-Pinilla et al., 2014, 2020b) and form functional heteromers with cross-antagonism as a fingerprint (Martínez-Pinilla et al., 2014). In heterologous systems, CB1 receptor modulates GPR55 signaling (Kargl et al., 2012), and because both receptors are also expressed in nigral terminals, the probability of a heteromeric interaction therein is high. GPCR–GPCR interactions are conformation-dependent and contribute to the stabilization of specific signaling states; receptor activation has been shown to facilitate these interactions (Goupil et al., 2012; Sharrocks et al., 2025).

Here, we investigated whether CB1-GPR55 heteromers are present in striatonigral neurons and their terminals, and examined their effects on cAMP accumulation, GABA release, and motor behavior. We also tested whether the order and timing of receptor activation influence heteromer formation/stabilization and function. Our findings show that CB1/GPR55 heteromers are expressed in striato-nigral terminals and that only sequential activation of CB1 followed by GPR55 in the substantia nigra pars reticulata, with a 16-minute interval, enhances receptor co-immunoprecipitation, cAMP accumulation, GABA release, and motor function compared with GPR55 stimulation alone. The results indicate that CB1 receptor activation promotes dimerization and/or stabilization of the CB1/GPR55 heteromer, thereby amplifying downstream cellular response and motor behavior.

## Materials and methods

### Animals

Male Wistar rats (200–250 g) housed together (five per cage) with food and water *ad libitum* and kept under a natural light cycle were used throughout. The rats were pretreated with reserpine (10 mg kg^–1^, i.p.) for 18 h before the preparation of slices to avoid the effects of endogenous dopamine. This treatment causes more than 92% of the decrement of the endogenous dopamine content in the substantia nigra (Nava-Asbell et al., 2007), and it is used to prevent the activation of dopamine receptors during depolarization with high K^+^ (Jijón-Lorenzo et al., 2018). Rats used for behavior PLA or immunohistochemistry experiments were not treated with reserpine. All procedures followed by the National Institutes of Health Guide for Care and Use of Laboratory Animals. They were approved by the Institutional Animal Care Committee of the CINVESTAV, making all efforts to minimize animal suffering.

### Kainic acid lesions

Animals were anesthetized with ketamine/xylazine (80/10 mg/kg i.p.) and mounted in a stereotaxic apparatus. Two microinjections of a kainic acid (KA) solution (1 μg/1 μL freshly dissolved in sterile saline, pH 7.4) were administered unilaterally into the dorsal striatum with a Hamilton syringe at coordinates: AP: 1.9, L: −2.2, P: −5, and AP: 0.9, L: −3.0, P: −4.0 according to the Atlas of Paxinos and Watson (1997). After injection, the needle was maintained in place for a further 5 min. Rats received a 5% glucose solution daily subcutaneous injection for 5 days. Fifteen days after the lesion, animals were sacrificed to dissect the striatum and nigral slices of the control and lesioned sides to PLA.

### Proximity ligation assay coupled with immunofluorescence

The obtaining and processing of the rat brain was carried out according to the protocol established by Sánchez-Zavaleta et al. (2022). PLA coupled with IF was performed according to Pacchiana et al. (2014) with slight modifications using a free-floating method. Coronal sections (30 μm) containing the dorsolateral striatum (Str) or substantia nigra pars reticulata (SNr) were incubated with citrate buffer for antigen retrieval (3 min). Subsequently, samples were treated with 2% donkey serum, 0.1% Triton X-100, and 1% bovine serum albumin in PBS 0.1M for 2 h at room temperature. In order to visualize Substance P-positive cells with CB1-GPR55 heterodimers, mouse anti-substance P monoclonal antibody (ab14184, 1:200), rabbit-anti-GPR55 polyclonal antibody (bs-7686R, 1:100) and goat-anti-CB1 polyclonal antibody (MBS422809, 1:100) were coincubated for 48 h at 4°C. After three PB washes, slices were incubated concomitant with the secondary antibody for substance P (Alexa 488 anti-mouse, 1:200), and secondary DNA probes: Anti-Goat PLUS and Anti-Rabbit MINUS (DUO92105-Sigma Duolink Bioscience) for 2 h at 37°C under gentle agitation (all reagents were diluent in *Duolink antibody diluent)*. Then, the samples were rinsed in buffer A at room temperature and were incubated with ligation solution and ligase for 1 h at 37°C. After washing with buffer A, samples were incubated with the amplification solution and polymerase for 100 min at 37°C under gentle agitation. Samples were washed with buffer A, followed by buffer B and by another wash with buffer B (1:100 diluted in PB). Finally, slices were washed and mounted in coverslips with a Vectashield^®^ mounting medium and DAPI.

For PLA protocol without IF, incubation with mouse anti-substance P monoclonal antibody and secondary Alexa antibody was omitted. The rest of the protocol was carried out as previously described. In addition, two negative controls were added: and antibody for D4 dopamine receptors (Sigma-Aldrich, AB1787P) instead of GPR55, because it has been reported that CB1 do not form heterodimers with D4 (Callén et al., 2012); and a second condition without primary antibodies.

### Laser confocal microscopy

Images were acquired with a Leica TCS SP8 confocal microscope (Leica Microsystems, Mannheim, Germany) equipped with an apochromatic 63 × oil immersion objective (NA 1.4), with a 405 nm laser and a laser that emits wavelength in the range of 470−670 nm. A slice per animal was selected for immunoreactivity analysis (6–7 animals/experimental group). 12−15 Z stacks with a 1.5 μm *z*-interval were acquired for each field. Channels were processed individually after getting the maximal projections of each image stack. PLA points were analyzed with ImageJ 2.1.0/Java 1.8.0 with the Nucleus Counter plugin according to the protocol reported by López-Ramírez et al. (2020). Once selected this threshold, value was applied uniformly for all images. In order to count and characterize all objects in the image thresholding, the built-in macro “Analyze particles” was applied. Objects larger than 3 μm^2^ were rejected. An example of an area evaluated to PLA count in striatum, or SNr, is shown in Supplementary Figure 1.

### Preparation of brain slices and synaptosomes

After rapid decapitation, the brains were removed and immersed in ice-cold artificial cerebrospinal fluid (aCSF), with the following composition (mM): 118.25 NaCl, 1.75 KCl, 1 MgSO_4_, 1.25 KH_2_PO_4_, 25 NaHCO_3_, 2 CaCl_2_, and 10 D-glucose. The brain was glued to a metal cube mounted on a Petri dish filled with ice-cold aCSF, and brain slices (300-μm thick) containing the substantia nigra pars reticulata were obtained with a vibroslicer (Campden Inc., Cambridge, United Kingdom). The slices were transferred to cold slides, and under a stereoscopic microscope, the SNr was microdissected and used in the form of slices to [^3^H]GABA release experiments or homogenates to obtain synaptosomal fractions.

Synaptosomal fractions were isolated from nigral slices from 10 rats. We used the method described by Alexander (1995) and adapted by Rangel-Barajas et al. (2011). Briefly, the slices were homogenized in buffer (sucrose, 0.32 M; HEPES, 0.005 M, pH 7.4), and then homogenates were centrifuged at 800 g for 10 min. The resulting supernatant was further centrifuged at 20,000 g for 20 min. From this second centrifugation, the supernatant (S1) was discarded, and the pellet (P1) was suspended and settled in sucrose 0.8 M, HEPES 0.005 M, buffer (pH 7.4), and newly centrifuged at 20,000 g during 20 min. Finally, the supernatant was discarded, and the new pellet (P2) containing synaptosomes was employed.

### Coprecipitation and western blot

Synaptosomes from SNr were incubated with RIPA buffer (sodium orthovanadate 1 mM, sodium pyrophosphate 10 mM, sodium fluoride 100 mM, glycerol 10%, Triton X-100 1%, Tris–HCl 50 mM, NaCl 150 mM, MgCl2 1.5 mM, EGTA 1 mM, SDS 0.1%, and sodium deoxycholate 1%), protease inhibitors (Complete tablets, Roche Applied Science, Mexico) and phenylmethanesulfonyl fluoride (PMSF): 1 mM. Total protein was quantified by the Bradford method. The samples with 250 μL of K-H were placed in Eppendorf tubes and the drugs were added in 10 μL volume. Incubation continued for 15 min for the first drug and 15 min plus to the second drug.

A sample of 350 μg of protein for coimmunoprecipitation was incubated with antibodies against CB1R (1:200) or GPR55 (1:200) for 12 h at 4°C. Then, A/G-agarose beads were added (Roche Applied Science, México) for the next 12 h. Then, samples were washed by centrifugation three times (10,000 rpm) for 5 min with 500 μL of RIPA buffer. The resultant pellet of the third centrifugation was resuspended in sample buffer (Glycerol 50%, Tris–HCl 125 mM, SDS 4%, Bromophenol blue 0.08%, b-mercaptoethanol 5%) and heated at 100°C for 10 min.

Samples and input (35 μg of protein for input) were loaded into the gel and proteins were resolved by SDS-PAGE at 120 volts for 120 min and transferred onto nitrocellulose blotting membranes (Amersham TM) and blotted for 2 h at room temperature in Tris buffered saline containing 5% non-fat dry milk. Membranes were incubated overnight at 4°C with antibodies CB1R (1:1,000) or GPR55 (1:1,000). Membranes were incubated with the respective HRP-conjugated secondary antibody: goat anti-rabbit IgG H&L (HRP, 1:40,000) (ab6721) or mouse anti-goat IgG H&L (HRP, 1:40,000) (sc-2354). Proteins were detected by chemiluminescence using ECL (Plus Amersham).

### [^3^H]cAMP accumulation

We carried out [^3^H]cAMP accumulation assays according to the method described by Rangel-Barajas et al. (2011). Synaptosomal fractions were incubated with [^3^H]-Adenine (130 nM) for 1 h at 37°C. After this time, they were suspended in buffer Krebs-Henseleit (K-H) with the following composition: NaCl, 127; KCl, 3.73; MgSO4, 1.18; KH2PO4, 1.18; CaCl_2_, 1.8; HEPES, 20; glucose, 11; and 3-isobutyl-1-methylxanthine (IBMX) 1 (all in mM). The samples with 250 μL of K-H were placed in Eppendorf tubes and the drugs were added in 10 μL volume. Incubation continued for 15 min for the first drug and 15 min plus to the second drug, and stopped by adding 100 μL of ice-cold trichloroacetic acid (30%) containing non-labeled ATP (2.5 mM) and cAMP (4.5 mM). After 20 min on ice, the supernatant was loaded onto Dowex 50W-X4 (300 μL per column). A fraction containing [^3^H]-ATP was eluted with 3 mL of distilled water. A second eluent obtained with 5 mL of distilled water was directly loaded onto neutral alumina columns. Alumina columns were finally eluted with 4 mL of 50 mM Tris-HCl buffer pH 7.4 to obtain [^3^H]-cAMP. ATP and cAMP eluents were transferred to vials, and radioactivity was determined by scintillation counting. The results were expressed as the ratio of [^3^H]- cAMP × 100/[^3^H]-cAMP + [^3^H]-ATP.

### [^3^H]GABA release

We determined [^3^H]-GABA release through methods described in detail by Jijón-Lorenzo et al. (2018). SNr slices from rats were pooled and left equilibrating for 30 min in aCSF, maintained at 37°C, and gassed with O2/CO2 (95:5 vol./vol.), then were incubated for 30 min in aCSF containing 80 nM [^3^H]-GABA (95 Ci/mmol). The labeling and perfusion solutions had amino-oxyacetic acid (10 μM) to prevent degradation of the label by GABA transaminase. At the end of this period, the excess label was removed by washing twice with ice-cold aCSF containing 100 μM of nipecotic acid to prevent the uptake of [^3^H]-GABA. Nipecotic acid was also included in all solutions used in all the following experiment steps. For each experimental group, slices were apportioned randomly between the chambers (80 μL, 20 superfusion chambers in parallel) and perfused at a 0.5 mL/min rate. Each chamber contained three slices, which were perfused with normal aCSF for 30 min before collecting fractions. To depolarize the terminals, the [K^+^] in the perfusion solution was increased to 15 mM (composition in mM: 101.25 NaCl, 13.75 KCl, 1 MgSO4, 1.25 KH2PO4, 25 NaHCO3, 2 CaCl_2_, and 10 D-glucose). Perfusates were collected in a fraction collector every 4 min. We determined the total amount of tritium remaining in the tissue at the end of the experiment. The slices were collected, treated with 1 mL of HCl (1M), and allowed to stand for 1 h before adding the scintillator.

[^3^H]GABA release was expressed initially as a fraction of the total tritium remaining in the tissue. The effect of drugs on the basal release of [^3^H] GABA was assessed by comparing the fractional release in fraction 2 (immediately before exposure of the tissue to the drug) and fraction four (immediately before exposure to 15 mM of K^+^), using an unpaired *t*-test. Changes in depolarization-induced [^3^H]GABA release by drugs and treatments were assessed by comparing the area under the appropriate release curves between the first (fraction 5) and last fraction collected (fraction 10) after the change to high K^+^, assuming that the basal release of [^3^H]GABA remained unchanged at the level measured in the fraction immediately preceding K^+^ stimulation. Previously, we found that when the release was determined in fraction 10 without any treatment, it was 98 ± 2% of that in fraction 4, indicating a minimal change in basal release.

### Drugs

Aminooxiacetic acid, AAOA; IBMX (3-isobutyl-1-methylxanthine); Arachidonyl-20-chloroethylamide hydrate, ACEA; ATP (adenosine 5-triphosphate disodium salt hydrate); ChTx from *Vibrio cholereae*; PTx from Bordetella pertussis; CID16020046 4-[4,6-dihydro-4-(3-hydroxyphenyl)−3-(4-methylphenyl)−6-oxopyrrolo[3,4-c]pyrazol-5(1H)-yl]-benzoic acid}; LPI (L-α-lysophosphatidylinositol sodium salt from Glycine max); Kainic acid (2-carboxy-3-carboxymethyl-4-isopropenylpyrrolidine); Adenine, [2,8-3H]−, > 97%, 1 mCi (37MBq), [3H] Adenine; Aminobutyric Acid (GABA) γ-[2,3-3H(N)]−, Specific Activity: 25–40 Ci (925 GBq−1.48 TBq)/mmoL, 1 mCi (37 MBq) was purchased from Perkin Elmer (Waltham, MA, United States).

### Circling behavior

Male Wistar rats, weighing 200–250 g, were anesthetized with ketamine/xylazine (80/10 mg/kg, i.p.). Turning was the recorder placing the rat in a square arena from 90 cm × 90 cm and the recorder with a camera for 1 h. The number of turns per minute was recorded for every minute by a double-blind method. For microinjections, a guide cannula (22-gauge, 14 mm long) was placed in the SNr for microinjections. The coordinates used were −2.2 lateral, −1.9 anteroposterior, and −7.3 mm from the dura for the right side and 2.2 lateral, −1.9 anteroposterior, and −7.3 mm from the dura for the left side, with an inclination of 34° to the interaural line to access the SNr. The cannula was secured with dental acrylic glued to the skull and small stainless-steel screws; a wire stylet was inserted into the cannula to prevent clogging. Rotational behavior was tested 3 days after surgery. The rat was gently restrained, and the stylet was replaced by an injection cannula (30-gauge, 14.5 mm long) connected to a 5 μL Hamilton syringe. Then, the animals were returned to the observation chamber. Drugs or vehicles were applied in a volume of 1 μL delivered for 2 min, and the cannula remained in place for three additional minutes. Finally, the animals were killed by an overdose of ketamine/xylazine, and the brains were removed and fixed in 10% formalin for 24 h. Brain sections (150 μm) were obtained, and injection sites were assessed by the cannula’s location. Experiments with cannula locations not corresponding to substantia nigra reticulata were discarded.

### Statistical analysis

All data were analyzed using GraphPad Software (San Diego, CA, United States) version 9.4.1. Except where indicated, the one-way ANOVA, combined with Tukey’s test, was used to compare differences between experimental conditions in the same experiment. An unpaired *t*-test was used to compare the two experimental conditions from different experiments or to compare only two experimental conditions within the same experiment.

## Results

### CB1/GPR55 heteromers are expressed in striato-nigral neurons and their terminals

**FIGURE 1:**
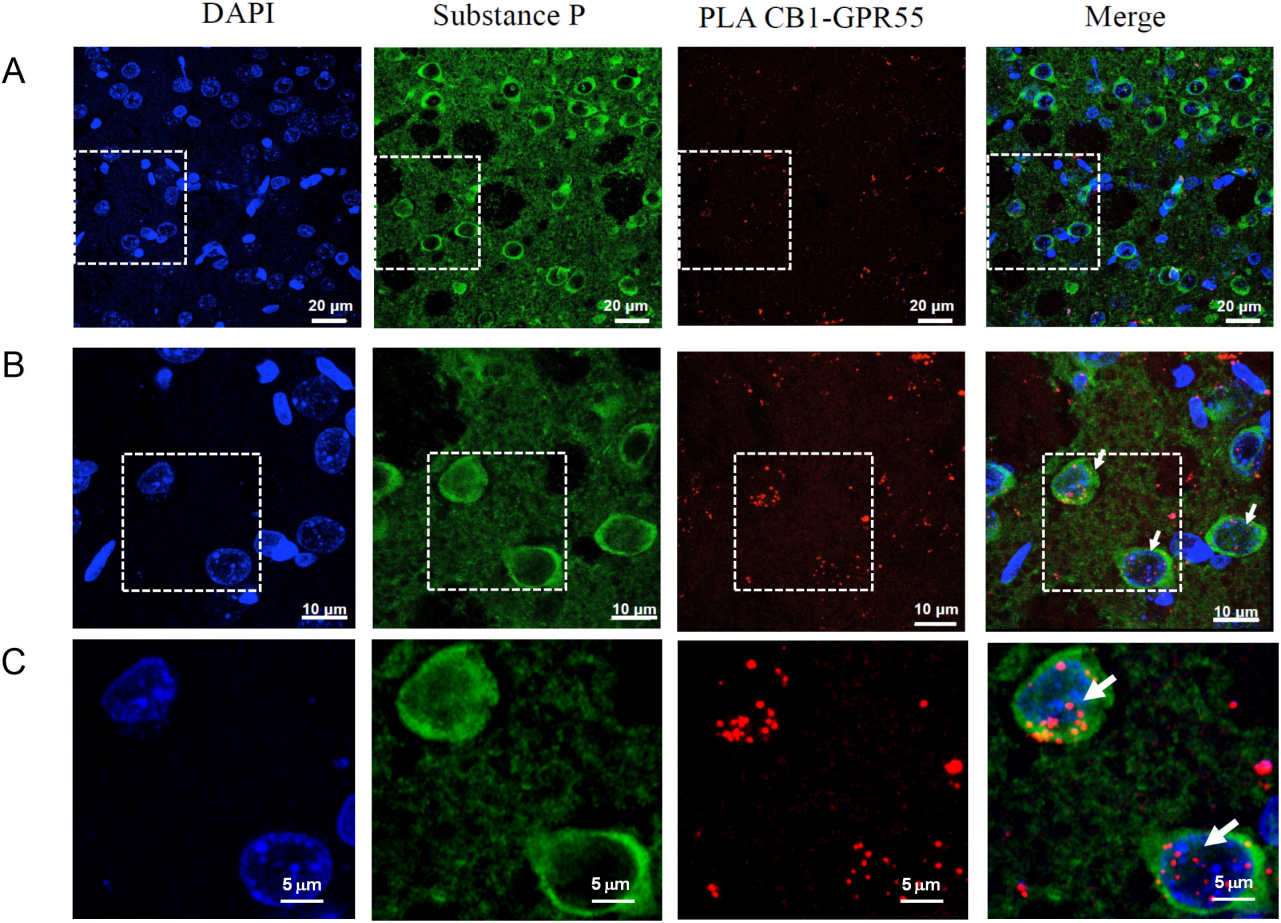
CB1/GPR55 heteromers are expressed in substance P-positive elements in the dorsal striatum. **(A)** Representative microphotographs of the immunohistochemical location of DAPI stain for nuclei (blue), Substance-P (red stain), PLA-dots for CB1/GRP55 receptors (red stain), and the merge of stain channels. **(B,C)** A magnification of upper microphotographs in which white dots indicate the association of PLA-dots and substance-P positive neurons.

**FIGURE 2:**
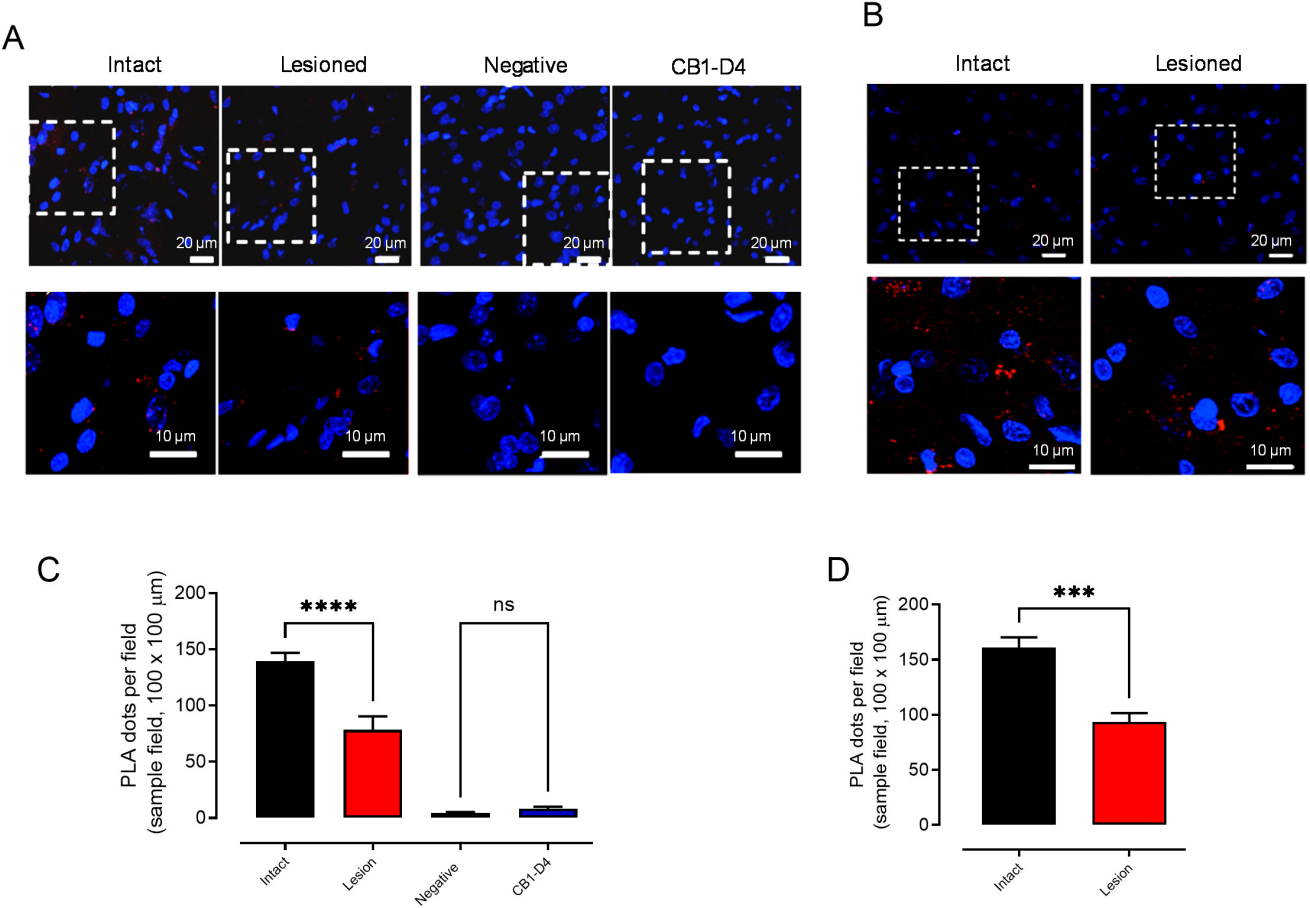
Expression of CB1/GPR55 heteromers in the soma and terminals of striatonigral neurons. **(A,B)** Present representative microphotographs of PLA dots in the dorsal striatum from both the intact and lesioned sides of rats that were unilaterally injected with kainic acid in the dorsal striatum. The images also include a negative control as well as results for PLA of CB1 and D4 receptors. In graphs **(C,D)** the count of PLA dots for CB1/GPR55 is shown in samples from 100 × 100 μm fields in the dorsal striatum of four lesioned rats, along with counts from the negative control and PLA of CB1/D4 receptors from four additional rats. The dot count comparison between the lesioned and intact sides is presented in panel **(D)**. In panels **(B,D)**, data are expressed as mean ± SEM, with statistical significance indicated as ****p < 0.0001, ***p < 0.001, and ns for not significant differences among groups. Data from panel **(C)** were analyzed using ANOVA followed by Tukey’s test, while **(D)** data were analyzed using an unpaired *t*-test with *n* = 8 analyzed fields from four rats.

We first assessed the expression of CB1/GPR55 heteromers in striatal neurons that project to the substantia nigra pars reticulata. We performed proximity ligation assays (PLA) in striatal and nigral slices, which were subsequently processed for immunofluorescence using substance P as a marker for striato-nigral neurons (Lee et al., 1986). In Figure 1A, PLA dots (in red) are localized in the dorsal striatum. Figures 1B,C show a magnified view, indicating that these dots are associated with substance P-positive neurons (green stain) and with non-positive cells. Figure 2A illustrates that following a kainic acid lesion in the striatum, the number of PLA dots decreased. The count of PLA dots per field reveals a nearly 50% decrease in the lesioned area compared to the intact, non-lesioned side (Figure 2C: mean PLA dots on the intact side = 139 ± 9 vs. lesioned side = 78 ± 12; *p* < 0.0001; *n* = 8; ANOVA followed by Tukey’s test). Moreover, the kainic acid lesion in the striatum also results in a significant reduction of PLA dots in the substantia nigra reticulata, though approximately 40% (Figures 2B,D: mean PLA dots on the intact side = 160 ± 9 vs. lesioned side = 93 ± 8; *p* = 0.0003; *n* = 6; Unpaired *t*-test).

### Coactivation of CB1 and GPR55 produces different effects on [^3^H]GABA release

**FIGURE 3:**
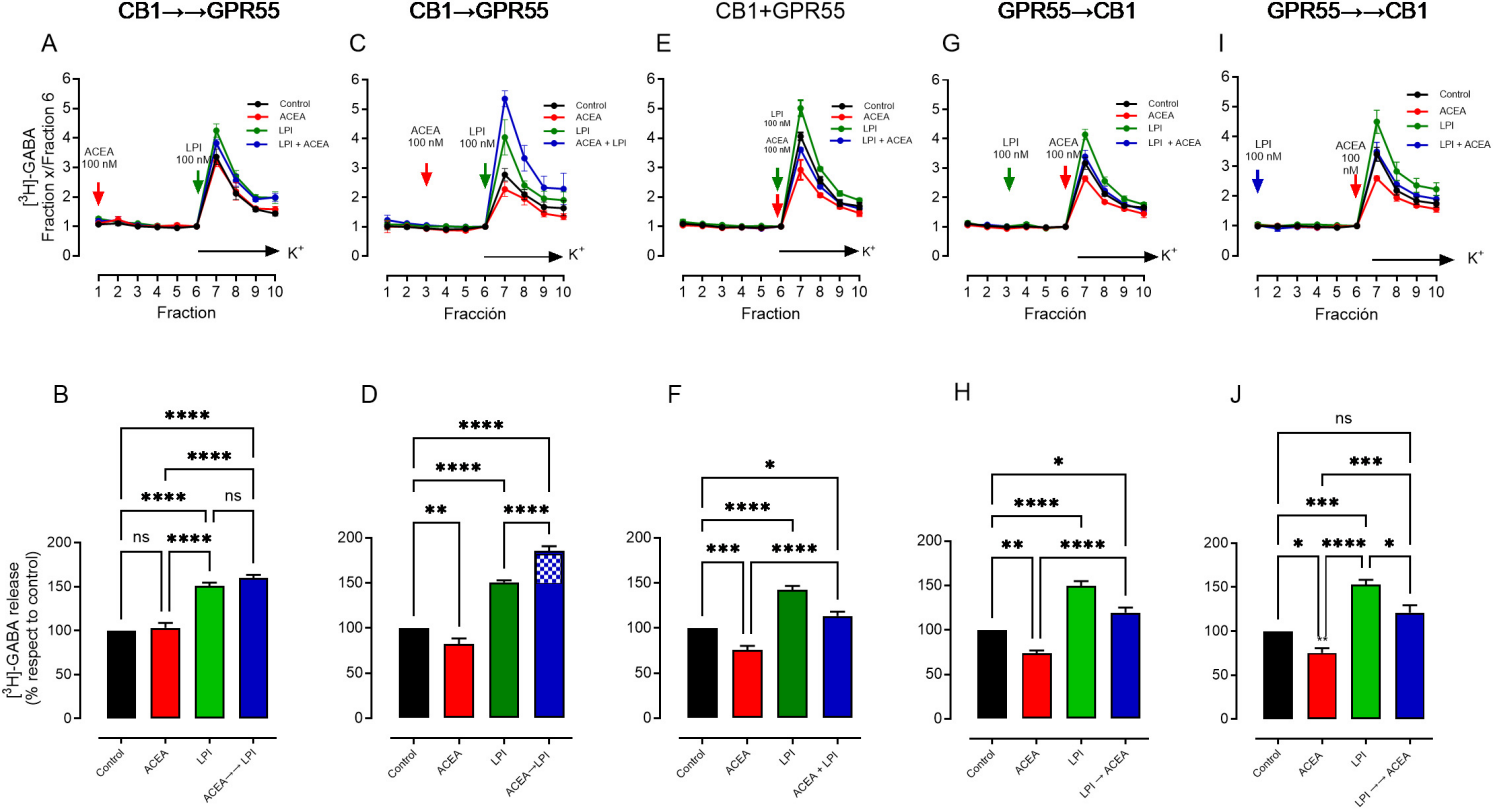
Different effects of coactivation of CB1 and GPR55 on GABA release depend on the activation sequence and timing. Representative experiments on [3H]GABA release are shown, illustrating the effects of activating CB1 first followed by GPR55, or vice versa, as well as simultaneous coactivation. The time intervals between activations were 24 minutes (→→), 12–14 min (→), and simultaneous activation (+). Selective agonists ACEA (100 nM) and LPI (100 nM) were used as indicated in panels **(A,C,E,G,I)**. The lower bar panels **(B,D,F,H,J)** present the percentage change in the area under the curve from the upper graphs. Statistical significance is indicated as follows: *p < 0.05, **p < 0.01, ***p < 0.001, ****p < 0.0001; ns indicates no significant differences between groups. A total of five experiments for each graph were conducted, and the results were analyzed using ANOVA followed by Tukey’s test.

To investigate the effects of CB1 and GPR55 heteromer activation on GABA release at striatonigral terminals, we conducted experiments where the order and timing of receptor activation using selective agonists were varied. Typically, in coactivation protocols, the agonist drugs are added simultaneously with or previously to the depolarization using high K^+^ medium (Figure 3E) and present throughout the experiment. In Figure 3A, the CB1 receptor agonist ACEA (100 nM) was applied starting from the first basal fraction, while LPI was introduced to activate GPR55 in the sixth fraction simultaneously with depolarization, resulting in a 24-min difference in coactivation, denoted as CB1→→GPR55 in the figure. When slices were treated with ACEA alone for 24 min before high K^+^, GABA release did not change significantly compared to the control (Figure 3B, red bar: GABA release control at 100% vs. ACEA at 103 ± 6, *p* = 0.934, *n* = 6, One-Way ANOVA followed by Tukey’s test). In contrast, GPR55 activation with LPI before high K^+^ stimulation, as previously reported (Sánchez-Zavaleta et al., 2022), significantly increased GABA release compared to the control (Figure 3B, green bar: GABA release control at 100% vs. LPI 151 ± 4, *p* < 0.0001, *n* = 6, One Way ANOVA followed by Tukey’s test). The coactivation of both receptors increased GABA release, which was not significantly different from the effect of LPI alone (Figure 3B, blue bar: GABA release LPI 151 ± 4% vs. LPI + ACEA at 160 ± 4%, *p* = 0.394, *n* = 6, One-Way ANOVA followed by Tukey’s test).

In the experiments depicted in Figures 3C,D, the activation of CB1 receptors with ACEA commenced in the third fraction, yielding in a 16-min delay in before LPI administration, as indicated by the arrow in the figure (CB1→GPR55). Unlike the experiment shown in Figure 3B, the activation of the CB1 receptor alone resulted in a significant inhibition of GABA release (Figure 3D, red bar: GABA release control 100% vs. ACEA 82 ± 6%, *p* = 0.0062, *n* = 6, One-Way ANOVA followed by Tukey’s test). As expected, GPR55 activation alone resulted in a similar increase in GABA release, as observed in Figure 3B (Figure 3D, green bar: GABA release control at 100% vs. LPI 151 ± 2, *p* < 0.0001, *n* = 6, One-Way ANOVA followed by Tukey’s test). Remarkably, the coactivation of both receptors resulted in a significant increase in GABA release compared to both the control and the LPI-only groups, indicating a potentiation of LPI effects. The checkered section of the bar graph represents the increase in GABA release produced by sequential activation with ACEA followed by LPI, compared to LPI alone (Figure 3D, GABA release with LPI is 151 ± 2% vs. ACEA + LPI, 186 ± 5%, *p* < 0.0001, *n* = 6, One-Way ANOVA followed by Tukey’s test).

Figures 3E,F, shown that separated activation of CB1 and GPR55 produced similar results as in Figure 3D, but coactivation with no time differences (indicated as CB1 + GPR55) reduces the GABA release stimulated by GPR55 receptor activation (Figure 3F blue bar, GABA release LPI 142 ± 4% vs. ACEA + LPI 113 ± 5%, *p* = 0.0002, *n* = 6, One Way ANOVA followed by Tukey’s). Thus, the potentiated effect of CB1 receptor activation on GPR55-mediated GABA release, observed in experiment 3D, changes to an apparent antagonistic effect when receptors are activated simultaneously (Figure 3F). In the experiments of Figures 3G–J, the activation of GPR55 by LPI was shifted, mirroring the experiments of Figures 3A–D, respectively, indicating GPR55→CB1 and GPR55→→CB1. It can be observed that the activation of receptors separately, as well as their coactivation, produced results similar to those observed in coactivation at the same time, as shown in Figures 3E,F.

### Sequential coactivation of CB1→GPR55 increases CB1/GPR55 immunoprecipitation

**FIGURE 4:**
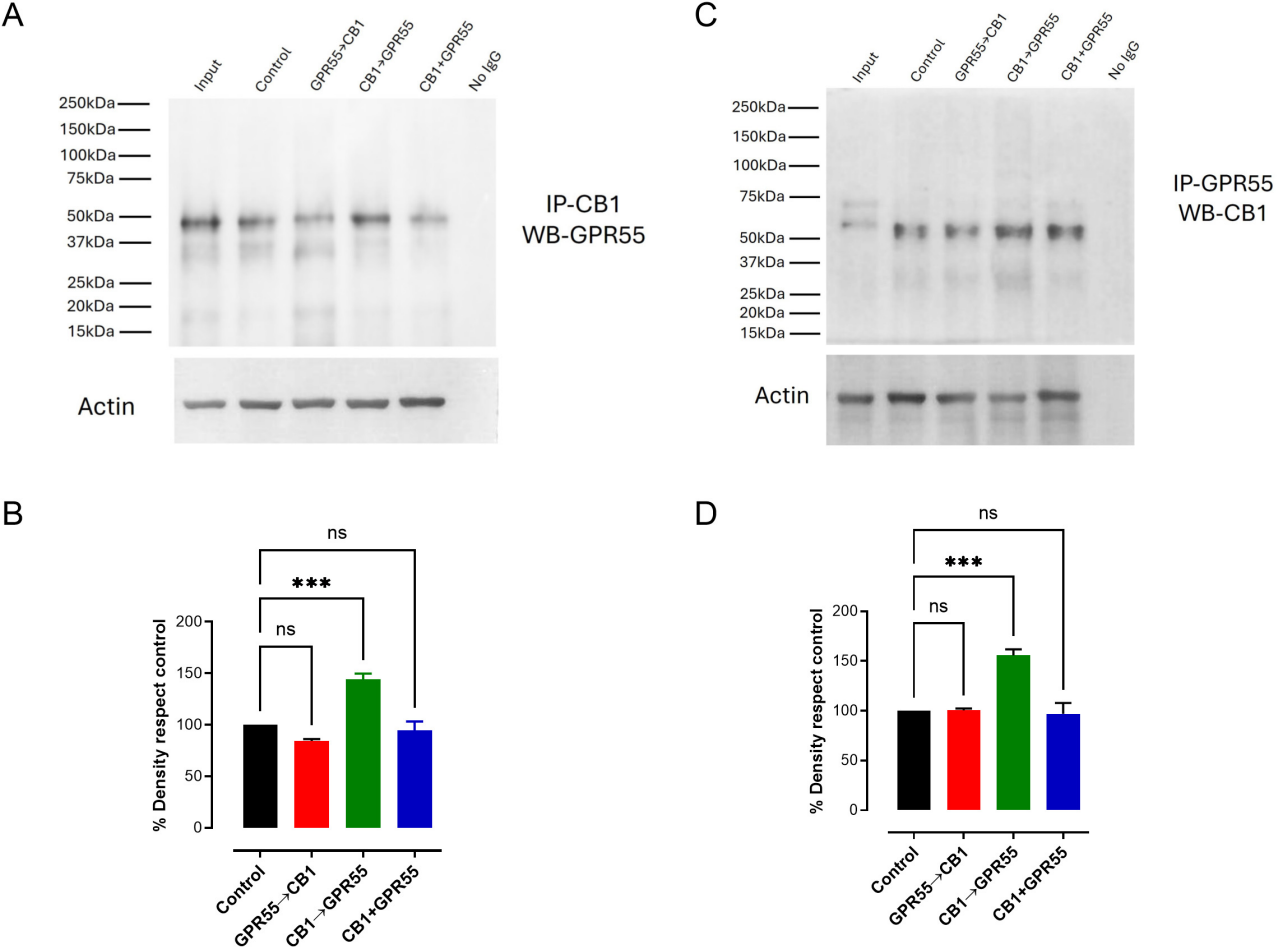
Sequential activation of CB1→GPR55 promotes an increase in immunoprecipitation. **(A,B)** Show typical coimmunoprecipitation experiments in which GPR55 coprecipitates with CB1 receptors and vice versa, respectively, in synaptosomes exposed to different sequences of receptor coactivation. **(C,D)** Show the densitometry analysis of 3 experiments. It can be observed that only CB1→GPR55 sequential coactivation increases by 50% the basal immunoprecipitation. ***p < 0.001 and ns, not significantly different from control, *n* = 3, ANOVA followed by Tukey’s test.

Since CB1 and GPR55 form heteromers at striato-nigral neurons (Figure 1) and CB1→GPR55 potentiate GABA release over GPR55 activation alone, we tested if receptors coprecipitate and whether sequential coactivation potentiates it. Nigral synaptosomes maintained *in vitro* were treated with CB1 agonist ACEA and GPR55 agonist LPI in GPR55→CB1, CB1→GPR55, and CB1 + GPR55 sequences of activations, after processing protein for immunoprecipitation of GPR55 and WB of CB1 and the inverse maneuver. In Figures 4A,C, representative blots and Figures 4B,D densitometry analysis of 3 experiments, it can be observed that CB1→GPR55 coactivation increases immunoprecipitation over the control and GPR55→CB1 and CB1 + GPR55 activations, as densitometry analysis indicates (Figure 4B, CB1→GPR55 density with respect to control 144 ± 5, *p* = 0.0003, *n* = 3; Figure 4D, CB1→GPR55 density with respect to control 155 ± 6, *p* = 0.0003, *n* = 3; One-way ANOVA, followed by Tukey’s). These data suggested an increment of CB1/GPR55 dimerization during CB1→GPR55 sequential activation.

### CB1→GPR55 G protein-coupled and signaling

GPR55 receptors stimulate the release of GABA at striatonigral terminals by activating adenylate cyclase (AC), leading to the production of cyclic adenosine monophosphate (cAMP) (Sánchez-Zavaleta et al., 2022). To investigate whether CB1 receptor activation enhances the effects of GPR55 on GABA release, we measured cAMP accumulation experiments during the coactivation of CB1 and GPR55 under three conditions: CB1→GPR55, CB1 + GPR55, and GPR55→CB1.

**FIGURE 5:**
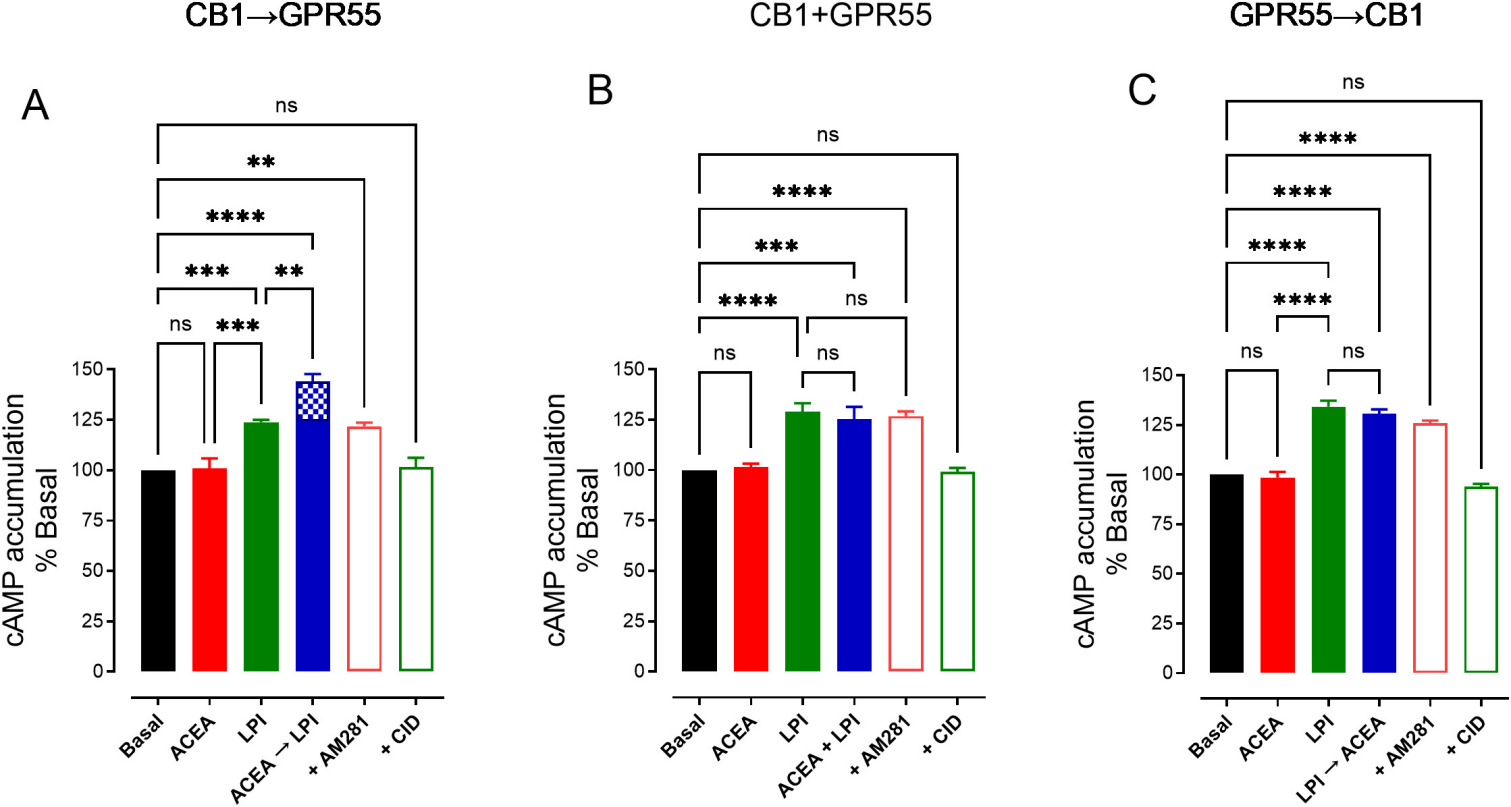
Effects of receptor interactions: CB1→GPR55, CB1 + GPR55, and GPR55→CB1 on cAMP accumulation. The graphs show the effects of coactivation of these receptors in synaptosomes from the substantia nigra pars reticulata of rats. **(A)** The effect of the sequential activation of CB1 leading to GPR55 is shown, with a time difference of 12–14 min. **(B)** The simultaneous activation of both CB1 and GPR55, while **(C)** presents the effects of GPR55 leading to CB1, again with a 12-min time difference. Statistically significant results are indicated as **p < 0.01, ***p < 0.001, and ****p < 0.0001, while ns denotes no significant differences between groups. A total of five experiments were conducted for each graph, and the results were analyzed using ANOVA followed by Tukey’s test.

In Figure 5A, it can be observed that the sequential activation of CB1 followed by GPR55 (CB1→GPR55) significantly increases cAMP accumulation, exceeding both the basal level and the level induced by lysophosphatidylinositol (LPI) (LPI-induced cAMP accumulation: 124 ± 2% vs. ACEA + LPI: 144 ± 3%, *p* = 0.0025, *n* = 5, ANOVA followed by Tukey’s test). Notably, the activation of the CB1 receptor did not modify cAMP accumulation (Basal cAMP accumulation: 100% vs. ACEA 101 ± 1%, *p* > 0.99, *n* = 5, ANOVA followed by Tukey’s test). Blocking CB1 with its selective antagonist, AM281, reduced cAMP levels of CB1→GPR55 to those observed with GPR55 activation alone (LPI-induced cAMP accumulation: 124 ± 2% vs. ACEA + LPI + AM281: 121 ± 2%, *p* = 0.99, *n* = 5, ANOVA followed by Tukey’s test). Furthermore, the addition of the GPR55 receptor antagonist CID16020046 completely inhibited the increase in cAMP induced by coactivation of CB1→GPR55 (ACEA + LPI cAMP accumulation: 144 ± 3% vs. ACEA + LPI + CID16020046 101 ± 4%, *p* < 0.0001, *n* = 5, ANOVA followed by Tukey’s test).

Consistent with the observations on GABA release, the enhanced cAMP accumulation observed during CB1→GPR55 activation was absent in during CB1 + GPR55 and GPR55→CB1, where CAMP levels did not differ from LPI alone (Figure 5B, ACEA + LPI cAMP accumulation: 125 ± 6% vs. LPI 129 ± 4%, *p* = 0.97, *n* = 5; Figure 5C, ACEA + LPI cAMP accumulation: 131 ± 2% vs. LPI 134 ± 3%, *p* = 0.804, *n* = 5, ANOVA followed by Tukey’s test). Additionally, the blockade of CB1 receptors with AM281 did not alter the effect of CB1→GPR55 coactivation, whereas the GPR55 antagonist CID16020046 prevented the increase in cAMP accumulation.

GPR55 stimulates GABA release through the cAMP→PKA pathway, but it does not involve Gs or Gi proteins (Sánchez-Zavaleta et al., 2022); it is likely to involve Gq (Xia et al., 2025). When G protein-coupled receptors form heteromers, they can alter the signaling pathways utilized by their individual receptors (Ferré, 2015) and exhibit a characteristic fingerprint (Franco et al., 2008; Martínez-Pinilla et al., 2014). In our experiments on GABA release, we evaluate whether the coactivation of CB1→GPR55 during heteromerization leads to a change in G protein coupling and exhibits the pharmacological fingerprint for the CB1/GPR55 heteromers, namely, cross-antagonism (Kargl et al., 2012).

**FIGURE 6:**
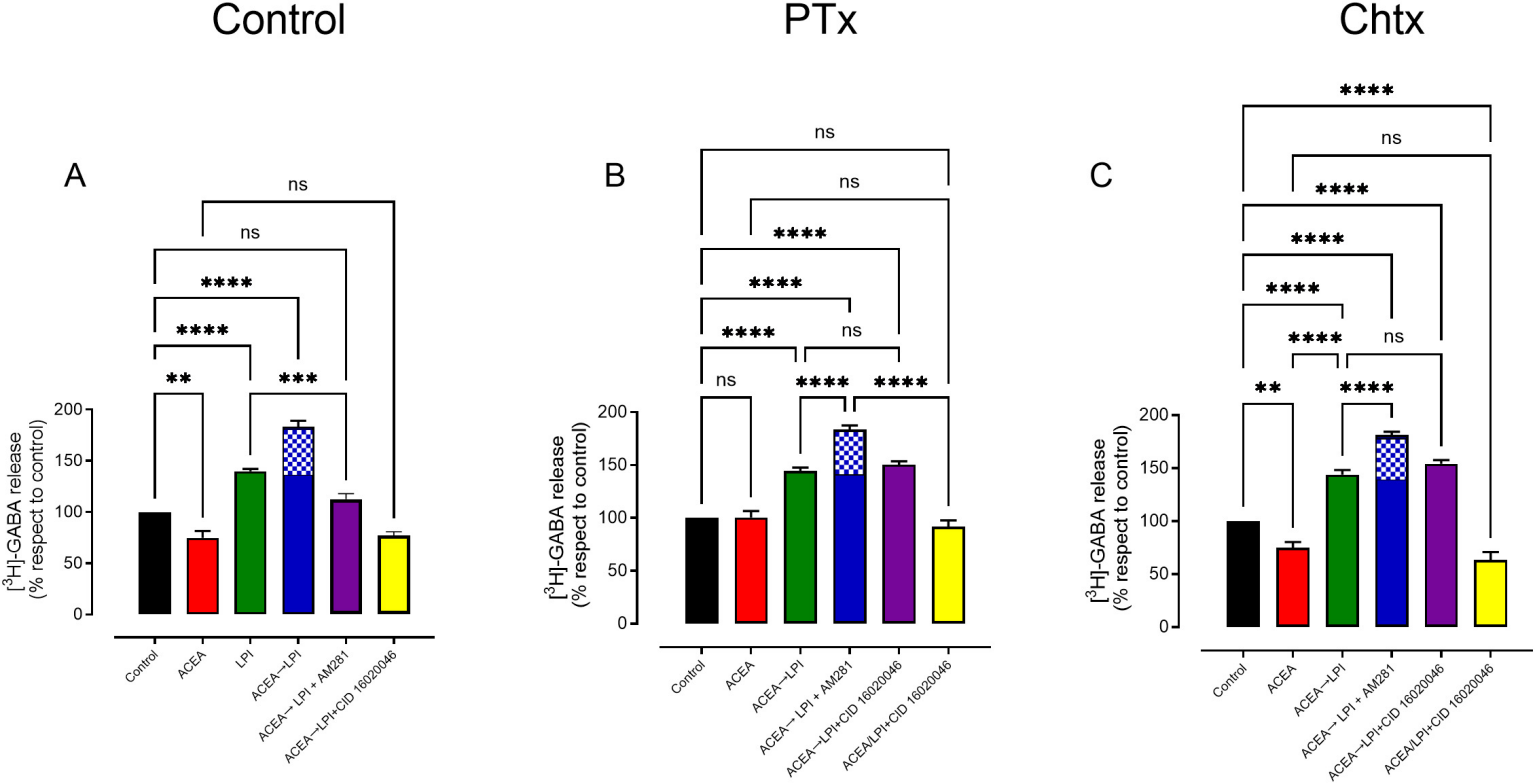
Pertussis toxin (PTx) and cholera toxin (ChTx) treatments did not alter the effects of CB1→GPR55 coactivation on GABA release in the substantia nigra pars reticulata. **(A)** The control effect of CB1→GPR55 coactivation on nigral GABA release, along with the antagonistic effects of AM281 (10 nM for CB1) and CID16020046 (10 nM for GPR55). **(B)** The slices were pretreated with PTx to assess the impact of blocking Gi proteins, while **(C)** examines the sensitization of Gs protein due to ChTx treatment. Significant levels are noted as **p < 0.01, ***p < 0.001, and ****p < 0.0001, while ns indicates no significant differences between groups. A total of four experiments were conducted for each graph, and the results were analyzed using ANOVA followed by Tukey’s test.

In CB1→GPR55 coactivation, the CB1 receptor potentiates cAMP and GABA release elicited by GPR55 alone. The increase in cAMP or GABA release over GPR55 stimulation likely reflects the effect of heteromerization (Figures 5A, 6A, blue bar, segment checkered). Figure 6A shows that the CB1 antagonist AM281 blocks this potentiation, reducing GABA release during CB1→GPR55 coactivation to levels similar to GPR55 activation alone (GABA release LPI 140 ± 2% vs. ACEA + LPI + AM281 129 ± 6% *p* = 0.0816, *n* = 4, ANOVA followed by Tukey’s). Similarly, the GPR55 antagonist CID16020046 decreases release of CB1→GPR55 coactivation, below the level induced by CB1 activation alone (GABA release ACEA 75 ± 7% vs. ACEA + LPI + CID16020046 77 ± 3% *p* = 0.7180, *n* = 4, ANOVA followed by Tukey’s).

The role of Gi protein in CB1→GPR55 activation was investigated by pretreating slices with PTx (see Materials and methods). As shown in Figure 6B, PTx treatment abolishes the ACEA effect on GABA release (control for GABA release is 100% vs. ACEA at 100 ± 4%, *p* = 0.943, *n* = 4, ANOVA followed by Tukey’s test). Additionally, PTx treatment did not change the effect of GPR55 activation alone or the coactivation of CB1 and GPR55 (refer to Figure 6B for green and blue bars). Notably, the blockade of the CB1 receptor with AM281 eliminated the increase in GABA release induced by CB1→GPR55 coactivation, reducing it to levels comparable to those of LPI alone (GABA release for ACEA→CB1 + AM251 is 150 ± 3, compared to LPI at 144 ± 3, *p* = 0.943). Interestingly, blocking GPR55 with CID16020046 during CB1→GPR55 coactivation completely prevented the effects of this coactivation. Finally, in Figure 6C, it is shown that pretreating slices with cholera toxin (ChTx) did not modify any of the effects of the added drugs during CB1→GPR55 activation.

### Behavioral effect of intranigral CB1→GPR55, CB1 + GPR55, and GPR55→CB1 activations

**FIGURE 7:**
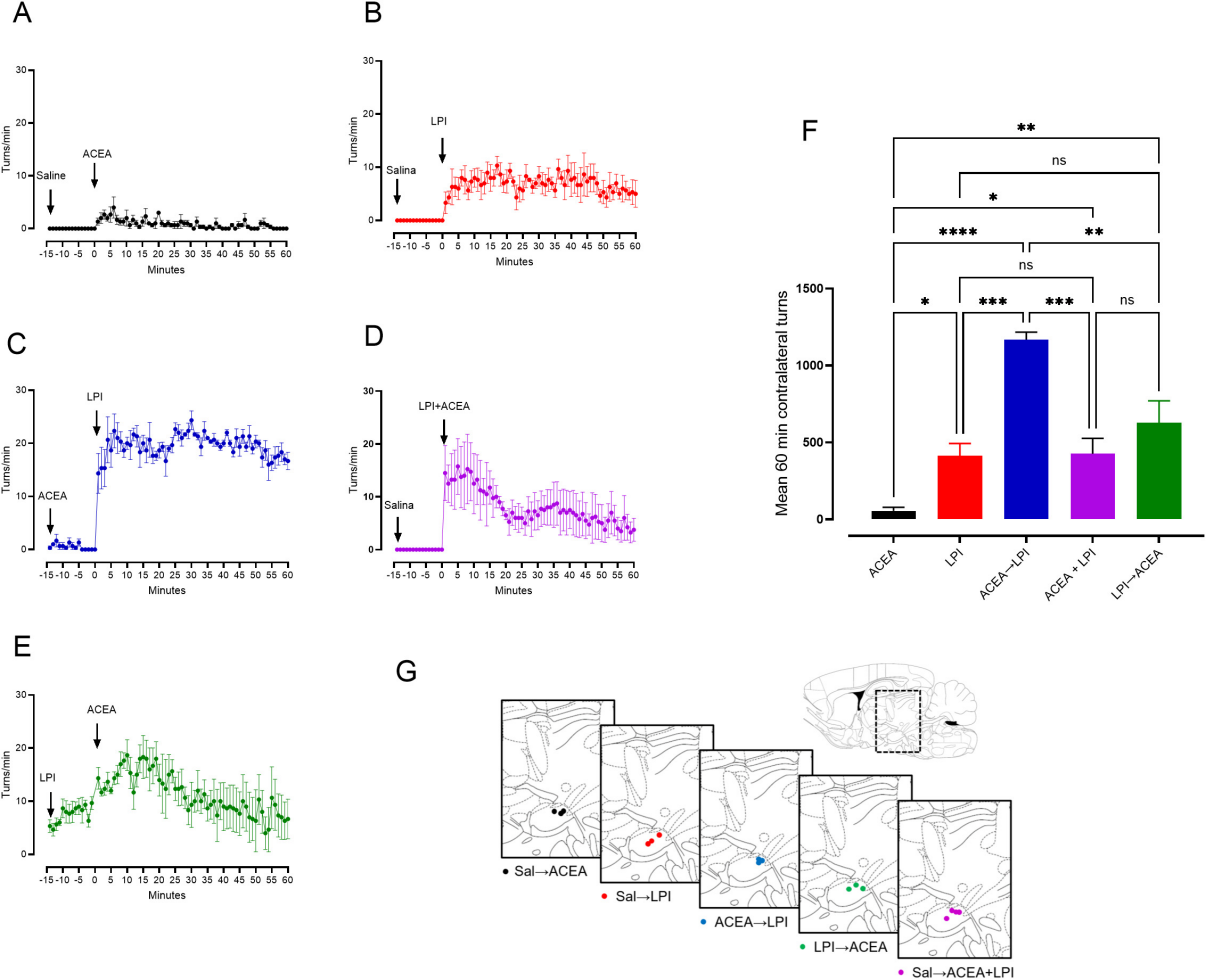
CB1 receptors enhance the stimulation of motor behavior through GPR55 during sequential coactivation of CB1→GPR55. The effects of intranigral injections of ACEA (17 ng/1 μl) to stimulate CB1 receptors and LPI for GPR55 (34 ng/1 μl) on circling behavior are illustrated in panels **(A,B)** respectively. These injections were preceded by saline administration 12–14 min prior to the drug injection. The sequential coactivations of CB1→GPR55, CB1+GPR55, and GPR55→CB1 are depicted in panels **(C–E)** respectively. The time interval between drug administrations in panels **(C,E)** was also 12–14 min. **(F)** Presents a graph showing the total number of contralateral turns over 60 minutes, analyzed using ANOVA followed by Tukey’s *post hoc* test. Significance levels are indicated as follows: *p < 0.05, **p < 0.01, ***p < 0.001, ****p < 0.0001, with “ns” indicating no significant differences (*n* = 3 rats). **(G)** Shows the approximate location of the cannula in the substantia nigra according to the Paxinos and Watson rat atlas.

Intranigral activation of GPR55 stimulates locomotor activity by increasing GABA release, independent of dopamine or the D1 receptor (Sánchez-Zavaleta et al., 2022). We now studied the effects of the proposed sequential activation of CB1→GPR55, CB1 + GPR55, and GPR55→CB1 using unilateral intranigral injections of agonists on motor activity, as evaluated by turning behavior. In Figures 7A,B, the effect of the injection of ACEA (17 ng/1 μL) and LPI (34 ng/1 μL) respectively into the substantia nigra is shown, it can be observed that the CB1 agonist ACEA, produces a slight contralateral turn during the 60 min period of evaluation (Figure 7F black bar, mean 55 ± 23 turns/60 min, *n* = 3 rats), and that LPI produces a significant contralateral turn as expected (Figure 7F red bar, mean 413 ± 79 turns/60 min, *n* = 3 rats). The sequential activation CB1→GPR55 by sequential injection of receptor agonists, produced a robust turning as shown in Figure 7C (Figure 7F blue bar, mean 1,168 ± 48 turns/60 min, *n* = 3 rats) over the effect of the injection of LPI alone (Figure 7F, LPI 413 ± 79 turns/60 min vs. ACEA→LPI 1,168 ± 48 turns/60 min, *p* = 0.0019, *n* = 3, ANOVA followed by Tukey’s). Also, can be observed that sequential activations of CB1 + GPR55 (Figure 7D, violet bar) and GPR55→CB1 (Figure 7E, green bar), did not produce more turns than observed by GPR55 alone activation by LPI (Figure 7F, LPI 413 ± 79 turns/60 min vs. LPI→ACEA 627 ± 142 turns/60 min, *p* = 0.266; and vs. ACEA + LPI 592 ± 229, *p* = 0.347, *n* = 3, ANOVA followed by Tukey’s). Note that in Figure 7E, the injection of LPI induces turning prior to the injection of ACEA. This effect is expected from the sole activation of GPR55, as shown in Figure 7B, but CB1 activation by ACEA did not significantly alter the total number of turns relative to GPR55. Figure 7G shows the approximate location of the cannula in the substantia nigra according to the Paxinos and Watson rat atlas.

## Discussion

Our data indicate that CB1 and GPR55 receptors can form heteromers at striatonigral terminals. This dimerization is increased by CB1 receptor activation at a critical time point. Dimerization of CB1 and GPR55 modulates GPR55 signaling, enhancing activity. This interaction increases cAMP formation and GABA release, thereby stimulating motor activity.

### CB1/GPR55 heteromers in striatonigral terminals

Several reports have indicated the presence of GPR55 receptors and CB1/GPR55 heteromers in the striatum (Marichal-Cancino et al., 2017; Celorrio et al., 2017; Martínez-Pinilla et al., 2014, 2020a; Ryberg et al., 2007). The functional consequences of GPR55 activation in the projections to the substantia nigra have been described (Sánchez-Zavaleta et al., 2022). However, the possible presence of CB1/GPR55 heteromers in this projection area has not been studied. In our study, we identified a clear association between the substance P (SP)-positive elements in the dorsal striatum and the signals from the proximity ligation assay (PLA) for CB1 and GPR55 receptors (Figure 1), indicating the presence of the heteromers in these neurons. Since the natural projections of SP-positive neurons lead to the substantia nigra (Hong et al., 1977) and the main location of CB1 receptors is at the nerve terminals (Mátyás et al., 2006), we evaluated the presence of heteromers in the substantia nigra using the PLA, similar to our analysis in the striatum. Notably, both regions exhibited similar levels of dots per area (Figures 2C,D), and striatal kainic acid lesions resulted in a comparable decrease of about 40–50% in both nuclei. Both structures express more dimers in other elements, as indicated by the specificity of the PLA assay, which showed no specific dots in the negative control and confirmed that PLA for CB1 and D4R does not form dimers (Callén et al., 2012). The glial or neuronal source of those heteromers remains to be elucidated. Therefore, according to our results, CB1/GPR55 dimers are expressed in SP-positive striatal neurons and their projections to the substantia nigra.

### The CB1/GPR55 functionality at striatonigral terminals

Our findings, illustrated in Figure 3, show that the timing and order of co-activation of CB1 and GPR55, using their respective selective agonists (Kargl et al., 2012; Oka et al., 2007), result in three distinct patterns of GABA release compared to the stimulation of GPR55 alone. GABA source in the substantia nigra, can be attributed mainly to striatal afferences (Misgeld et al., 2007), less from pallidal projections (Smith and Bolam, 1989), and a few from local axon collaterals (Deniau et al., 2007). After striatal kainic acid lesions, GABA uptake decreases by nearly 70% (Schwarcz and Coyle, 1977), indicating that the major capture sites in nigra are located in striatal terminals; thus, GABA release determined in our experiments comes mainly from striatal terminals.

Coactivation of CB1 →→GPR55 produces GABA levels similar to those achieved with GPR55 activation alone (Figures 3A,B). Although we would expect an antagonistic interaction based on the reports of the effects of the receptors on GABA release separately, our data, which activate CB1 alone with ACEA, indicate no effect on GABA release (Figure 3B, red bar). Prolonged receptor activation may lead to desensitization, limiting its effects on GABA release and membrane expression (Jin et al., 1999). This contrasts with the effect of GPR55 on GABA release, which stimulates release independently of the duration of activation, suggesting no apparent desensitization (Figures 3B,D,F,H,J). It has been reported that some presynaptic receptors are resistant to desensitization, thereby helping regulate neurotransmitter release (Liu et al., 2013). However, the presence of GRKs at nerve terminals indicates that such mechanisms exist (Arriza et al., 1992) and have been reported for presynaptic receptors (Gainetdinov et al., 2004). Although the activation of GPR55 by LPI at relatively high concentrations produces β-arrestin internalization in heterologous systems (Kapur et al., 2009; Oka et al., 2007; Yin et al., 2009), no such observation has been made in native systems; if internalization of GPR55 by the β-arrestin mechanism occurs, it does not discard intracellular signaling by the cAMP as has been shown for other GPCRs (Gurevich and Gurevich, 2019). Further research is necessary to explore the apparent lack of desensitization in more detail.

In coactivations of CB1 + GPR55 (3E and F), GPR55→CB1 (3G and H), and GPR55→→CB1 (3I and J), CB1 receptors produce an antagonistic effect on GABA release with respect to GPR55 activations, and receptors activated separately produce the expected effect. CB1 receptors have been reported to decrease GABA release (Chan et al., 1998), whereas GPR55 is known to increase it (Sánchez-Zavaleta et al., 2022). The antagonistic effect via different signaling pathways is highly probable, as the decrease in release observed during coactivation of the CB1 and GPR55 receptors, compared to GPR55 alone, is comparable to the decrease observed when CB1 is activated with ACEA relative to the control. For instance, in the experiments: in 3F LPI minus LPI + ACEA, the decrease was 29% compared to the control minus ACEA of 25%; in 3H LPI minus LPI + ACEA, the decrease was 30% compared to control minus ACEA at 27%; and in 3J LPI minus LPI + ACEA, it was 32% compared to control minus ACEA at 24%. Stimulation of GPR55 with LPI, when administered either just before depolarization (Figures 3B,D,F) or 12–14 or 24 min prior to depolarization (Figures 3H,J), increases GABA release with the same potency.

The most notable effect observed is the increased release of GABA during the simultaneous activation of CB1→GPR55 (see Figures 3C,D). The increase in GABA release from CB1→GPR55 receptor activation is more significant than that from GPR55 activation alone and also exceeds the control levels. The apparent paradox in Figure 3D, where ACEA alone suppresses GABA release, yet ACEA + LPI produces a potentiation beyond LPI alone, suggests that, at the time point of CB1→GPR55 sequential activation, two pharmacological signals coexist at the striatonigral terminals. On one hand, CB1 activation engages its canonical Gi/o-mediated inhibition of GABA release (Katona and Freund, 2012). On the other hand, when GPR55 is subsequently stimulated, the receptors appear to engage a heteromer-specific signaling that overrides CB1-induced suppression. This is consistent with evidence that GPCR heteromers shift the coupling preference and signaling efficacy of their individual monomers (Ferré, 2015). In this context, GPR55 activation may bias the CB1–GPR55 heteromer toward Gα12/13 and RhoA-dependent pathways (Oka et al., 2007), leading to enhanced intracellular Ca^2+^ and increased GABA release, as previously shown for GPR55 alone (Sánchez-Zavaleta et al., 2022). Taken together, the facilitation produced by CB1→GPR55 coactivation most likely reflects heteromer-specific signaling that outweighs the inhibitory effect of CB1 activation alone. This supports the notion that temporal ordering of receptor engagement does not sum the actions of each receptor but instead enables the formation/stabilization of a unique signaling pathway at striatonigral terminals.

### CB1 activation induces an increment in CB1/GPR55 heteromerization

The increase in GABA release during CB1→GPR55 activation is associated with a rise in the coprecipitation of the receptors at nigral terminals (Figure 4). The presence of the CB1-GPR55 co-IP signal without exogenous activation suggests that a portion of the CB1-GPR55 heteromers exist in a pre-assembled state. This interpretation is consistent with previous reports showing that CB1 forms heteromers with other GPCRs, such as the D2 dopamine receptor (Marcellino et al., 2008); μ-, κ-, and δ-opioid receptors (Navarro et al., 2008); A2A receptors (Carriba et al., 2007), and others.

On the other hand, the observed increase in CB1-GPR55 co-IP following sequential receptor activation could reflect either *de novo* heteromer formation or stabilization of pre-existing complex. Receptor-receptor interaction depends on the stabilization of specific receptor conformations, which can be induced by ligands (Goupil et al., 2012; Sharrocks et al., 2025). In this context, μ- and δ-opioid receptors (MOR and DOR, respectively) form transient heterodimers for brief periods of 120–180 ms, and they dissociate and rebind every few seconds; moreover, the selective MOR agonist DAMGO significantly increases MOR-DOR heterodimer lifetimes (Zhou et al., 2025). Under our experimental conditions, the addition of the CB1 agonists (ACEA) could promote the CB1-GPR55 heterodimer stabilization. This pattern suggests that the enhancement of heterodimerization or stabilization of the heteromer depends on CB1 receptor activation, as evidenced by the absence of increment of co-IP in other forms of coactivation (CB1 + GPR55 or GPR55→CB1; see Figure 4).

Finally, constitutive heteromer formation begins in the endoplasmic reticulum (Hasbi et al., 2007). Therefore, under our experimental conditions, in which receptors are activated for only 12–14 min, the novo heteromer formation in the endoplasmic reticulum and subsequent trafficking to the cell membrane are unlikely, for example, it takes approximately 2 h for delta opioid receptor to reach the plasma membrane (Petäjä-Repo et al., 2000). With our experimental design, we cannot confirm the novo formation. However, we have conducted preliminary PLA experiments after incubation with ACEA and ACEA/LPI for 30 min in primary cultures of MSN from the dorsolateral striatum. The results show an increase in PLA signal, suggesting enhanced heteromer formation or stabilization (Supplementary Figure 2).

In our preparation, it is possible that the recruitment of multiple CB1-GPR55 heterodimers, rather than the activation of GPR55 alone, is required to produce a measurable enhancement of GABA release observed in the nigrostriatal terminals within slices. This result aligns with previous evidence showing that GPCR heteromers can amplify or reconfigure intracellular signaling with respect to monomeric receptor activation (Ferré et al., 2025). The temporal window of receptor activation is determinant for the observed physiological effects. Prolonged CB1 receptor activation induces receptor desensitization and internalization via GRK/β-arrestin pathways (Nguyen et al., 2017), thereby reducing the probability of heteromer signaling. Conversely, brief activation may engage only isolated monomeric receptors.

The case of GPR55→CB1 coactivation suggests that GPR55 activation alone is insufficient to induce dimerization; instead, CB1 is the factor that promotes it. Thus, the signal that enhances GABA release during GPR55→CB1 coactivation likely originates from a heteromer-specific signaling state, rather than from the independent activation of either receptor alone. This explanation is supported by co-IP data, which show increased co-IP signal under this specific activation order. Altogether, our findings highlight a temporally sensitive and coordinated mechanism in which CB1 drives a heteromeric state with GPR55 capable of modulating GABA release.

Research on cAMP accumulation has enhanced our understanding of the dimerization that occurs during coactivation of CB1→GPR55 receptors. When GPR55 is activated at these terminals, it increases cAMP levels, which subsequently leads to the release of GABA at striatonigral terminals (Sánchez-Zavaleta et al., 2022). The coactivation of CB1→GPR55 produces higher cAMP levels than either CB1 + GPR55 or GPR55→CB1 activation, indicating that the dimers formed or stabilized during sequential receptor stimulation function through cAMP signaling, similar to GPR55 alone. Currently, coactivation effects are blocked by inhibition of the CB1 receptor with AM281, a selective CB1 antagonist (Lan et al., 1999). This is significant because activating the CB1 receptor alone with ACEA does not result in any change in cAMP accumulation (Figure 5A), indicating that CB1 receptors do not couple to cAMP, despite a decrease in GABA release (Figure 3D), which is probably mediated by the βγ subunit of the G protein (Walsh and Andersen, 2020). Additionally, the GPR55 antagonist CID16020046 prevents all effects on cAMP accumulation, suggesting that GPR55, whether alone or in a dimerized state with CB1, stimulates cAMP. Both AM281 and CID16020046 inhibit the dimerization signal, indicating a cross-antagonist effect and serving as evidence for the previously described CB1/GPR55 dimer (Kargl et al., 2012). Notably, in the other two forms of coactivation, CB1 + GPR55 or GPR55→CB1, there are no effects of cAMP accumulation or cross-antagonism. Cross-antagonism is also observed in GABA release. As shown in Figure 6A, AM281 prevented the effect of CB1→GPR55; however, the GABA level is lower than that produced by LPI alone. This can be explained by the fact that some of the CB1 receptors are not dimerized but are activated during drug administration. This is further confirmed by the observation that blocking GPR55 with CID16020046 prevents both GPR55 activation and the coactivation of CB1→GPR55 at control levels.

When we evaluated the effects of blocking Gi proteins using PTx pretreatment or sensitizing Gs with ChTx, we did not observe any modifications in the dimer signal, indicating that the signaling pathways were not coupled to these G proteins, as previously discussed for GPR55 (Sánchez-Zavaleta et al., 2022). It has been shown that CB1 receptors can couple to Gs proteins and stimulate cAMP (Abadji et al., 1999). The ChTx treatment did not unmask this couple, as it did not modify either the ACEA or ACEA + LPI effects on cAMP accumulation. This suggests that the signal in the CB1/GPR55 dimer originates from GPR55 and is potentiated by the CB1 receptor. Similar dimeric interactions have been observed in other CB1 receptor heteromers. For instance, in a dimer with the orexin-A receptor, CB1 activation enhances the increase in intracellular Ca^2+^ induced by orexin-A receptor activation and leads to increased immunoprecipitation of the receptors; this dimer also has cross-antagonism (Imperatore et al., 2016). In HEK293 cells transfected with the CB1/GPR55 dimer, CB1 receptor-mediated ERK activation is enhanced, although this occurs through a different mechanism of interaction (antagonistic); it also exhibits cross-antagonism. Moreover, despite the observed effects on the signal, the presence of cross-antagonists is considered a characteristic feature of the CB1/GPR55 dimer (Martínez-Pinilla et al., 2014).

### The functional motor response to the activation of CB1/GPR55 heteromers

Intra-nigral activation of GPR55 receptors enhances locomotor activity, as assessed by motor asymmetry (Sánchez-Zavaleta et al., 2022). The resulting circling behavior occurs contralateral to the injection site, indicating that increased motor activity at this location is due to a rise in GABA release (Kaakkola and Kääriäinen, 1980). Therefore, coactivation of CB1 and GPR55, which promotes GABA release more effectively than GPR55 alone, should result in enhanced circling behavior, as observed when comparing Figures 7B,C. Conversely, injections in which GPR55 is activated first, followed by CB1, or when both are activated simultaneously, did not induce greater circling than GPR55 activation alone (Figures 7D,E vs. Figure 7B). This finding indicates that the temporal sequence of receptor activation is critical for engaging heteromer-specific signaling that amplifies GABA release, leading to higher GABA release compared to other activation combinations. Taken together, these data demonstrate that CB1→GPR55 heteromer activation produces a distinct and functionally relevant modulation of nigral output, with clear behavioral consequences on motor asymmetry.

The requirement of 12–14 min of CB1 pre-activation before GPR55 stimulation to elicit maximal circling behavior suggests that heteromer-mediated neuromodulation is temporally gated, rather than instantaneous. CB1 receptors exhibit relatively slow activation/desensitization kinetics (Sim-Selley, 2003) and can progressively modulate intracellular signaling pathways, including cAMP, PKA, and ERK, over several minutes (Dalton and Howlett, 2012). A sustained period of CB1 activation may therefore be necessary to prime intracellular conditions, such as Gβγ availability, Ca^2+^ microdomains, or β-arrestin engagement before GPR55 activation. This stabilizes the heteromer and promotes a signaling shift that increases GABA release. Similar time-dependent requirements for GPCR heteromer activation have been observed in other systems, where receptor cross-talk evolves over extended periods and defines the functional output of the heteromeric complex (Hillenbrand et al., 2015; Kim et al., 2024).

Endocannabinoid are released during prolonged motor states such as sustained locomotion or postural maintenance (Kettunen et al., 2005). These conditions could promote CB1 receptor activation for extended periods of time before lipid mediators (such as LPI) reach effective concentrations to activate GPR55. The functional consequences of CB1/GPR55 heteromers at the striatonigral level are critical for motor coordination, as they modulate GABA release independently of dopamine. D1 receptors at striatonigral terminals significantly influence motor performance (Bergquist et al., 2003), and the loss of GPR55 has been shown to impair this function (Wu et al., 2013). A common feature of both receptors is their ability to stimulate the release of GABA, suggesting that CB1/GPR55 may amplify motor regulation driven by GPR55. In Parkinson’s disease, where motor performance is profoundly disrupted (Meredith and Kang, 2006), CB1/GPR55 heteromers levels increase in the caudade and putamen of MPTP-treated primates (Martínez-Pinilla et al., 2020b), supporting the idea that this heteromeric complex may serve as an adaptive compensatory mechanism. These findings suggests that targeting CB1/GPR55 heteromers may represent a promising therapeutic strategy, offering an intervention that is not dependent on dopaminergic mechanisms, as proposed by Dr. Franco’s group. Additionally, CB1/GPR55 heteromers may exert complementary neuroprotective effects, as supported by studies in cellular models of Parkinson’s disease (Martínez-Pinilla et al., 2019).

## Conclusion

CB1 receptor activation stabilizes CB1/GPR55 heteromers and potentiates GPR55 effects on cAMP accumulation, GABA release, and motor behavior in the rat substantia nigra. Targeting this heteromer pharmacologically could be a promising therapeutic strategy for Parkinson’s disease. However, several questions remain regarding our findings, including the mechanism of dimerization, the ratio of heteromers to individual receptors, the physiological activation of each receptor, and the characteristics of the heteromer, among other factors, that represent future directions.

## Data Availability

The raw data supporting the conclusions of this article will be made available by the authors, without undue reservation.
