# Supplementary figures and images for: Coactivation of CB1 and GPR55 promotes GABA release and motor behavior at striatonigral terminals through increased dimerization induced by CB1 activation
Source: Front Mol Neurosci. 2026 Feb 26;19:1717829. doi: 10.3389/fnmol.2026.1717829 (PMC12979432; doi:10.3389/fnmol.2026.1717829)

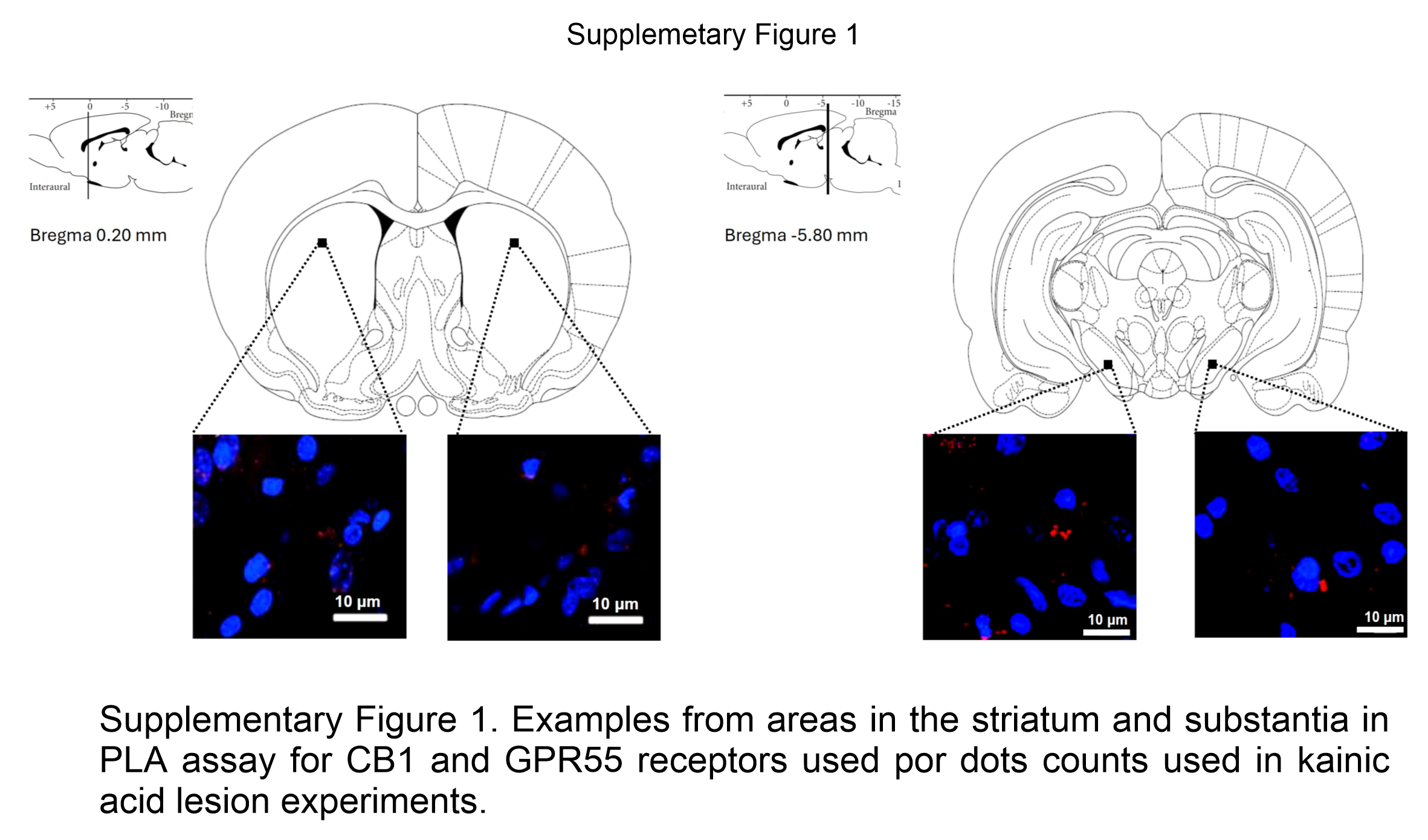

Supplement: Supplementary file 1 [file Image_1.tif]

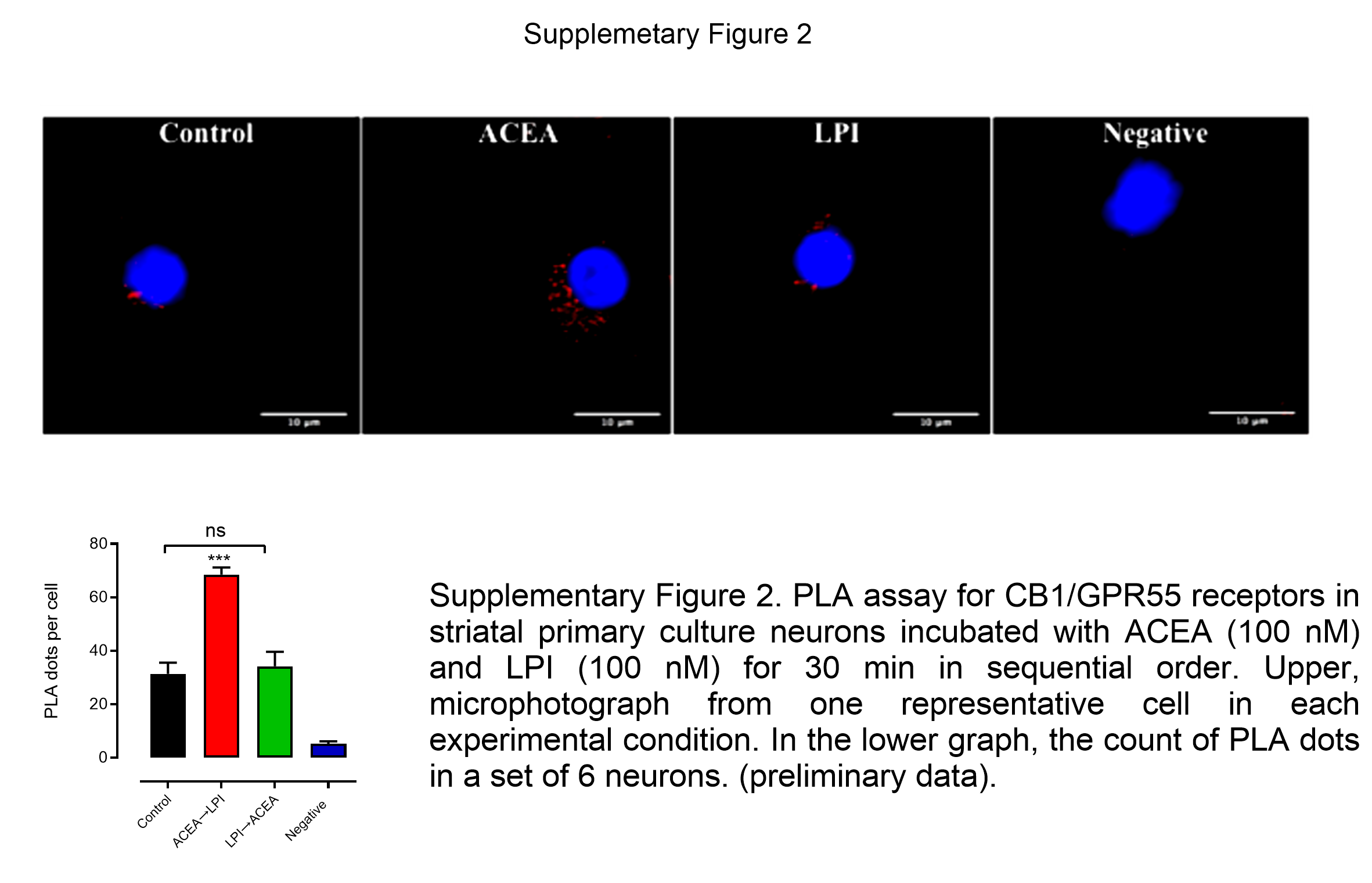

Supplement: Supplementary file 2 [file Image_2.tif]
